# A comparison of self‐report and discrepancy measures of camouflaging: Exploring sex differences in diagnosed autistic versus high autistic trait young adults

**DOI:** 10.1002/aur.2873

**Published:** 2022-12-09

**Authors:** Victoria Milner, Emma Colvert, William Mandy, Francesca Happé

**Affiliations:** ^1^ Social, Genetic and Developmental Psychiatry Centre Institute of Psychiatry, Psychology and Neuroscience, King's College London London UK; ^2^ Research Department of Clinical, Educational, and Health Psychology University College London London UK

**Keywords:** autism diagnosis, autistic camouflaging, autistic traits, female autism, sex differences

## Abstract

Camouflaging describes masking or compensating for autistic traits and/or related difficulties. Some evidence suggests autistic females camouflage more than autistic males, potentially contributing to delayed or missed diagnosis. Studies predominantly adopt self‐report measures of camouflaging, potentially reflecting a person's intent to camouflage without accurately measuring effectiveness (i.e., success in fulfilling the intended effect of minimizing the appearance of autistic traits) of camouflaging. Discrepancy scores between underlying cognitive difficulties (e.g., theory of mind) and observed autistic traits (henceforth camo_ToM_), or between self‐reported autistic traits and observed autistic traits (henceforth camo_SRS_), may provide a more accurate measure of camouflaging effectiveness. Three measures of camouflaging administered to autistic males (*n* = 46) and females (*n* = 40), and adults with equally high levels of autistic traits but no diagnosis (*n* = 45 males, *n* = 43 females) recruited from a large population‐based sample were compared. Self‐report measures of camouflaging were significantly correlated with camo_SRS_ scores only. Both discrepancy scores were correlated with each other. Adults with high autistic traits, but no diagnosis, had higher discrepancy camouflaging scores than diagnosed adults, but self‐reported scores were similar. Diagnosed females scored higher than diagnosed males across all camouflaging measures, but no sex difference occurred in the high trait group. This might indicate that autistic females have higher intentions and greater effectiveness when camouflaging, compared with autistic males. For camo_SRS_ only, high trait males scored significantly higher than diagnosed males; no group difference occurred for females. These results suggest that, despite all participants intending to camouflage to some extent, effective camouflaging as measured by discrepancy scores is higher in undiagnosed high autistic trait individuals. One interpretation is that effective camouflaging reduces the likelihood of autism diagnosis in males and females with high autistic traits.

## Lay Summary

Most studies use self‐report camouflaging questionnaires which measure “intention” to camouflage. One way to look at how effective a person's camouflaging is to look at the difference between autistic traits rated by another person and self‐reported autistic traits, or relevant cognitive difficulties. We used these three measures of camouflaging to look at differences between autistic males and females, and males and females without a diagnosis but with high levels of autistic traits. Our results suggest that participants in all groups report an intention to camouflage. When looking at the effectiveness of camouflaging, the participants with high traits but no diagnosis had higher scores. This suggests that effective camouflaging might make it less likely for both males and females to get an autism diagnosis. Females were also found to have higher camouflaging scores across all measures. This might indicate that autistic females have higher intentions and greater effectiveness when camouflaging, compared with autistic males.

## INTRODUCTION

Many autistic adults adopt strategies to mask their autistic traits or compensate for related difficulties, also known as camouflaging (Cook et al., 2021). Camouflaging strategies span a broad range of behaviors including suppressing stimming behaviors, rehearsing conversation starters before social interactions, or copying the body language, interests or speech of peers (Mandy, 2019). Strategies may be consciously learnt throughout a person's life or subconsciously adopted in response to difficult life experiences (Hull et al., 2017). A recent article proposes a shared framework with “impression management”—transactional and context‐dependent strategies that almost everyone will use at some point in their life (Ai et al., 2022). However, the underlying neurocognitive routes, motivations for, benefits and costs of camouflaging may be unique to autistic individuals (Ai et al., 2022).

Motivations for camouflaging are manifold, typically arising in situations of poor person‐environment fit, reflecting the fact that autistic people tend to occupy social settings mainly designed by and for nonautistic people (Mandy, 2019). Often, individuals camouflage in an attempt to obtain a goal such as facilitating more positive social interactions, success in school or the workplace, and/or to reduce instances of bullying and discrimination (Cage & Troxell‐Whitman, 2019; Hull & Petrides et al., 2020).

Anecdotal evidence and empirical literature consistently highlight the potential negative consequences of camouflaging. Hull et al. (2017), among others, have reported that camouflaging can sap large amounts of energy leading to exhaustion. Other studies have found that poorer mental health and quality of life are associated with more camouflaging (Ai et al., 2022; Cook et al., 2021). For instance, Milner et al. (2022) found that camouflaging scores measured on a self‐report questionnaire predicted lower psychological quality of life for autistic adults, and lower social quality of life for those with high levels of autistic traits but no formal diagnosis.

A further consideration is that camouflaging autistic traits may impact diagnostic experiences. Currently, an autism diagnostic process typically involves a clinical assessment, behavioral observation (such as with semistructured assessments) and developmental history gathered from the individual and/or informants close to the person (NICE, CG128;, 2019). It is possible therefore, that if an individual is able to camouflage their autistic traits within the behavioral observation (and indeed, when in the company of the informant), the clinician may not be able to recognize or understand the full extent of their autistic traits and any related difficulties, and thus may not make a diagnosis (Bargiela et al., 2016; Hull et al., 2017). The impact of camouflaging might extend even prior to reaching the diagnostic pathway, with poorer recognition of autistic traits from key stakeholders such as parents or teachers. Due to this, it is plausible (but debated; Fombonne, 2020) that there is a group of individuals who have high autistic traits, but no diagnosis, due to their ability (whether conscious or not) to camouflage. Livingston & Shah et al. (2019) gathered accounts from individuals who reported using camouflaging strategies, without a formal diagnosis of autism. They found that many participants endorsed similar themes to the participants with a formal autism diagnosis. Furthermore, the use of camouflaging strategies was identified by participants as one of the key contributors to delays in autism diagnosis (Livingston & Shah et al., 2019). These findings echo those of Hull et al. (2017), which showed delays and questioning of autism diagnosis due to participants not appearing autistic because of the use of camouflaging strategies. Lack of diagnosis can be a barrier to support and reasonable adjustments, as well as understanding of the self and from others (Ruiz Calzada et al., 2012); therefore, it is essential to ensure timely diagnosis for all.

The rise of research interest in camouflaging, within the last decade, largely stemmed from clinical observations and anecdotal evidence in the written work of autistic females (Sedgewick et al., 2021). Camouflaging has been posited as a barrier to diagnosis for autistic females, and a possible contributor to the disparity in autism diagnoses between males and females (Lockwood Estrin et al., 2021). The gender ratio is currently estimated to be 3:1 males: females receiving an autism diagnosis (Loomes et al., 2017). However, evidence also shows that autistic females wait longer and/or are less likely to receive a diagnosis, despite having the same level of autistic traits and number of clinical appointments as their autistic male peers (Begeer et al., 2013; Giarelli et al., 2010; Rutherford et al., 2016). Wing (1981) hypothesized that autistic females may be less likely to receive a diagnosis due to apparently adept social skills that may have been learnt. Yet, to date, there is insufficient empirical research evidence to determine whether camouflaging has a direct effect on the likelihood of obtaining a diagnosis for females versus males.

There is some evidence of sex differences in camouflaging of autistic traits. A systematic review of 29 papers quantitively measuring camouflaging identified 18 studies that examined sex/gender differences (Cook et al., 2021). Interestingly, the results of the majority of these studies (*n* = 12) suggested that females camouflage to a greater extent, across more contexts, more often and for longer than males (Cook et al., 2021). Conversely, six studies (e.g., Cage & Troxell‐Whitman, 2019) reported no differences in camouflaging between males and females in their samples (ns range = 92–262). No studies found that autistic males camouflage more than autistic females. One possible reason for the mixed evidence regarding sex differences and camouflaging autistic traits may be attributed to the measures used to operationalize camouflaging. Most studies (*n* = 13) used the self‐report Camouflaging Autistic Traits Questionnaire (CAT‐Q; Hull et al., 2019). The psychometric construct of the CAT‐Q has demonstrated measurement invariance for males and females (Hull et al., 2019), the CAT‐Q and similar self‐report measures (e.g., the social masking subscale of the Questionnaire for Autism Spectrum Conditions; Attwood et al., 2011) capture an individual's conscious camouflaging strategies (and, by implication, motivation), but not necessarily the degree to which these strategies are effectively used (i.e., the extent to which a person is perceived as less autistic) in everyday life.

An alternative approach to measuring camouflaging is to examine the difference between external, or observed, autistic behaviors, and internal, or underlying, cognitive differences/difficulties related to autism. The latter can be measured with objective tasks (e.g., tests tapping theory of mind [ToM]) or through self‐reported autistic traits (including nonobservable characteristics such as sensory sensitivities and thinking style). If the observed autistic behavior scores are lower than the relevant internal measure—either cognitive characteristics or how “autistic” a person feels—this may indicate effective use of camouflaging strategies. This approach was proposed by Livingston and Happé (2017) and operationalized by Lai et al. (2017) who created two discrepancy scores that were combined to measure camouflaging; the first was the difference between observed autistic traits measured by the Autism Diagnostic Observation Schedule (ADOS‐2; Lord et al., 2012) and scores on the Reading the Mind in the Eyes Task (Baron‐Cohen, Wheelwright, Hill, et al., 2001); and the second was the difference between ADOS‐2 scores and self‐reported autistic traits captured by the Autism Quotient (Baron‐Cohen, Wheelwright, Skinner, et al., 2001). This study found that, despite variability within groups, on average, females had significantly greater camouflaging scores than males (Lai et al., 2017). Therefore, it may be that, while autistic males report camouflaging to the same extent as autistic females in some studies, autistic females may on average camouflage more *effectively*. These findings were replicated by Schuck et al. (2019) in a sample of 17 autistic males and 11 autistic females. By contrast, Livingston & Shah et al. (2019) examined the discrepancy between ADOS scores and ToM task scores, categorizing participants as either “high” compensators (i.e., those who had low observer ratings and lower ToM ability, thus “appearing” less autistic despite underlying cognitive difficulties) or “low” compensators (those who had higher observer ratings and lower ToM ability). A similar distribution of males and females was found in both compensator groups; however, the sample was predominantly male (*n* = 112/136, 82%) and the study did not actively examine sex differences and may have been underpowered to detect these. Therefore, it is possible that adopting a discrepancy approach to examining camouflaging may yield more consistent evidence for sex differences in camouflaging autistic traits (Cook et al., 2021). In a study by Corbett et al. (2021) using a similar approach to operationalize “low” and “high” compensators, there was a larger proportion of males in the “low compensation” group compared with females. Whilst both cognition‐observable traits and self‐reported‐observable traits discrepancy scores yield unique benefits, it is important to consider the differences between these approaches (see Lai et al., 2021 for further discussion).

To date, to the best of our knowledge, no study has examined sex differences comparing the two predominant methods of measuring camouflaging: self‐report versus discrepancy scores. It is essential to compare these two measures to determine how related the two constructs are (Cook et al., 2021; Fombonne, 2020; Williams, 2022). Furthermore, no studies have investigated sex differences in self‐report and discrepancy measures of camouflaging between individuals with an autism diagnosis, and individuals with high autistic traits but no formal diagnosis of autism. The latter are a particularly important participant group as by including these participants, we potentially avoid the circularity of research examining potential barriers to diagnosis by including solely diagnosed samples. This study aimed to fill the gap in the literature and extend understanding of measures of camouflaging and potential sex and/or diagnostic group differences.

We predicted the following:

- Self‐reported camouflaging (CAT‐Q scores) will correlate positively with camouflaging discrepancy scores
- Females will show greater camouflaging than males across both self‐reported and discrepancy camouflaging scores
- There will be group differences in camouflaging scores between diagnosed autistic (Dx group) and high autistic trait participants (HT group); HT adults will camouflage more than Dx adults.

## METHODS

### Participants

A sample of 174 young adults was drawn from the third phase of the Social Relationships Study (SRStudy) which is a substudy of the Twins Early Development Study (see Colvert et al. (2015) for more information on recruitment to the SRStudy and supplementary material for details on the third phase). The third phase of the SRStudy specifically aimed to examine gender differences in a range of variables between those with an autism diagnosis and those with high autistic traits, but no formal diagnosis.

Participants were aged 21–24 years old (mean = 21.77, SD = 1.00) at the time of data collection. Data were available from 83 females (47.7%) and 91 males (52.3%). Note, participants' assigned sex at birth was used in these analyses.

In total, 86 participants had a formal diagnosis of autism (Dx; 49.4%) and 88 had high levels of autistic traits but no formal diagnosis (HT; 50.6%). Participants were assigned to the “high trait” group if they had not reported a formal diagnosis but scored above standardized cut off scores on the Childhood Autism Spectrum Test (Scott et al., 2002) at age 8 or 12 years (cut off score = 15), and/or the Social Responsiveness Scale—Second Edition (SRS2; Constantino & Gruber, 2012) (cut off raw score = 60) during the third phase of the SRStudy (at age 21–25 years). Within the diagnosed group, formal diagnosis was confirmed from parental report at earlier stages of the SRStudy; the average age of diagnosis for females was 12.6 (SD = 4.91) years, and for males was 9.3 years (SD = 5.57). Females were diagnosed significantly later than males in this sample (*t*(65) = 2.60, *p* = 0.006).

As the focus of this study was sex differences, participants were allocated to four groups: diagnosed females (*n* = 40), diagnosed males (*n* = 46), high trait females (*n* = 43), and high trait males (*n* = 45).

### Measures

#### Self‐reported camouflaging

**Camouflaging Autistic Traits Questionnaire** (Hull et al., 2019): to assess self‐reported camouflaging behavior all participants completed the CAT‐Q. At the time of data collection for this study, the CAT‐Q was still in development and as such a 32‐item version of the questionnaire provided by the author was used, as opposed to the 25‐item final version. For all questions, participants responded using a 7‐point Likert scale ranging from 1 “strongly disagree” to 7 “strongly agree” for all statements. The CAT‐Q authors were consulted during scoring of this measure and advised use of a total score summing scores for the 25 finalized items. Higher scores indicated greater reported use of camouflaging strategies.

#### Observer‐reported autistic characteristics

**Autism Diagnostic Observational Schedule** (Lord et al., 2012): the ADOS is the current gold standard observational measure of autism, used widely by clinicians and researchers. All participants completed module four of the ADOS‐2, suitable for adolescents and adults with fluent speech, with a trained researcher. Behavior and speech during the assessment is coded by the administrator and 16 items are included in a coding algorithm capturing the core diagnostic criteria for autism. The sum of all algorithm item scores was calculated and used in these analyses. Higher scores indicate a higher number of observed autistic behaviors.

#### Self‐reported autistic traits

**Social Responsiveness Scale (Second Edition)** (Constantino & Gruber, 2012): self‐reported autistic traits were assessed using the SRS2, a 65‐item questionnaire assessing social interest and ability. Each item on the SRS2 is rated on a 4‐point Likert scale from 1 not true to 4 almost always true. A summed score was computed and included in these analyses. Higher scores indicate higher levels of autistic traits.

#### Theory of mind

**Strange Stories Film Task** (Murray et al., 2017): in this task, participants watched a series of videos, during which two actors interacted with each other. Participants completed a practice trial at the beginning of the task. After each clip, participants were asked to explain why one character said something that was not literally true. Responses were rated for recognition of mental states on a scale of 0 (incorrect or no report), 1 (partial report), or 2 (accurate full report). A sum of scores is used in these analyses. Higher scores indicate greater ToM ability.

**Frith‐Happé Triangles Animations Task** (Abell et al., 2000): during this task, participants watched five video animations of two triangles moving around the screen. Following this, participants were asked what happened in the video. The first video was a “goal‐directed” animation used as a practice trial; the remaining four (scored) trials were “mental state” animations. The responses are rated on the report of mental states in their response on a scale of 0–5 (as described in Castelli et al., 2002). A sum of scores is used in these analyses. Higher scores indicate greater ToM ability.

As the two ToM measures were significantly correlated with each other (*r*(155) = 0.355, *p* < 0.001), a composite ToM score was created by computing an average of the two standardized ToM scores (see data analysis section). Composite ToM score was used throughout the analysis.

#### Intelligence quotient

**Wechsler's Abbreviated Scale of Intelligence—Second Edition** (WASI‐II; Wechsler, 2011): participants with verbal language completed the matrix reasoning and vocabulary tasks from the WASI‐II. Raw scores were converted into measures of verbal and perceptual reasoning. A full‐scale intelligence quotient (IQ) score was calculated for each participant.

**British Picture Vocabulary Scale ‐ Second Edition (**BPVS‐2; Dunn et al., 1997) **and Raven's Progressive Matrices** (Raven & Court, 1938). Non/minimally verbal participants were invited to complete the BPVS and Raven's Progressive Matrices tasks in place of the WASI‐II. The average of the scores from these two tasks were calculated as a full‐scale IQ score for each participant.

### Procedure

Ethical approval was obtained from Bromley NHS Research Ethics Committee (reference number: 16/LO/1472). Data were collected between 2016 and 2019 as part of a larger battery of tasks forming the third phase of the SRStudy. The ADOS‐2 and ToM measures were completed during face‐to‐face visits conducted either at the participant's home or at a research centre. The remaining measures were completed online at a time convenient to the participant either before or after the in‐person measures.

### Data analysis

The study was preregistered on the Open Science Framework (OSF) website (doi: 10.17605/OSF.IO/TFSN2).

All statistical analyses were conducted using IBM SPSS Statistics version 27.

All key variables (both ToM measures, SRS total score, ADOS‐2 total score) were standardized using a mean centering and scaling approach (similar to Lai et al., 2017). This approach allows all variables to be subtracted from each other to create meaningful discrepancy scores. The scaled ToM composite score was then transformed (each value multiplied by −1) so that the direction of scores was in line with the other key variables; thus, higher ToM score indicated poorer ToM, aligning with potentially more autistic traits.

Two discrepancy scores of camouflaging were calculated. The first camouflaging discrepancy score (henceforth camo_ToM_) was calculated as ToM (composite ToM score) minus observed autistic behavior (ADOS‐2 scores). The second camouflaging discrepancy score (henceforth camo_SRS_) was calculated as self‐reported autistic traits (SRS‐2 scores) minus observed autistic behavior (ADOS‐2 scores). For both discrepancy scores, due to mean centering and scaling, the scores range from 1 to −1. Higher scores indicate greater camouflaging.

The associations between self‐reported camouflaging and discrepancy scores were examined using correlational analyses; first with the whole sample, and then for males and females separately. As discrepancy scores were nonnormally distributed, nonparametric Spearman's correlations were conducted. The strength of the association between the camouflaging measures were compared between males and females using Fisher's *r*–*z* transformations computed with an online calculator (www.psychometrica.de/correlation.html).

A mixed 2 × 2 × 3 ANOVA was conducted to assess the effect of sex, diagnostic group, and measure type, on camouflaging score. The main effect of sex was examined to test the hypothesis that females would show greater camouflaging scores than males. The main effect of group was assessed to test the hypothesis that HT group would score higher for camouflaging than Dx group. The interaction between sex, group and measure type was assessed to determine whether there are greater sex differences for HT/Dx participants when using discrepancy measures, compared with self‐report measures. No a priori predictions were made for a three‐way interaction. Post hoc tests were used to examine two‐way interactions between variables. No a priori predictions were made for the two‐way interactions.

## RESULTS

A summary of key variable mean scores and distributions can be found in Table 1. Group distributions of standardized scores can be found in the supplementary material.

**TABLE 1:** Mean (SD) and range of scores split by group

	Dx		HT		Sig. group differences
	Female (*n* = 40)	Male (*n* = 46)	Female (*n* = 43)	Male (*n* = 45)	
	*Raw scores*				
CAT‐Q total (max = 175)	105.27 (24.71) 48–156	96.17 (23.24) 50–139	97.06 (29.79) 41–151	100.39 (19.17) 65–144	–
ADOS‐2 score (max = 32)	17.38 (8.56) 3–38	18.52 (9.64) 4–42	10.90 (8.71) 1–40	8.66 (7.39) 0–27	Dx > HT***
Triangles task (ToM intentionality) (max = 25)	14.00 (4.06) 6–21	14.12 (4.81) 0–24	14.69 (4.43) 1–21	15.77 (3.88) 1–22	–
Strange stories task (ToM mental state) (max = 12)	2.87 (2.07) 0–7	3.28 (2.00) 0–8	3.51 (1.83) 0–8	3.56 (1.59) 0–6	–
SRS score (max 195)	97.09 (22.81) 11–141	77.93 (24.12) 29–133	73.64 (25.56) 25–134	74.17 (20.17) 45–146	Dx F > Dx M*** Dx F > HT F***
IQ (max = 160)	97.26 (20.00) 26.25–132	94.83 (23.83) 22.61–126	95.68 (17.04) 49–128	102.35 (11.12) 81–131	–
	*Standardized camouflaging scores*				
CAT‐Q	0.03 (0.14) −0.30–0.32	−0.02 (0.13) −0.29–0.22	−0.02 (0.17) −0.34–0.29	0.00 (0.11) −0.20–0.25	–
Camo_ToM_	−0.05(0.13) −0.33–0.35	−0.06(0.13) −0.43–0.34	0.07(0.09) −0.19–0.28	0.07(0.10) −0.20–0.36	Dx < HT***
Camo_SRS_	0.00(0.13) −0.28–0.21	−0.08(0.19) −0.43–0.31	0.05(0.13) −0.25–0.28	0.06(0.12) −0.20–0.26	Dx F > Dx M*** Dx M < HT M***

*Note*: Significant sex/group differences are indicated in the last column.

Abbreviations: ADOS‐2, Autism Diagnostic Observation Schedule; CAT‐Q, Camouflaging Autistic Traits Questionnaire; Dx, diagnosed; HT, high trait; IQ, Intelligence Quotient; SRS, Social Responsiveness Scale; ToM, Theory of Mind.

*** *p* < 0.001.

### Correlations between camouflaging variables

Spearman's rho correlations within the whole sample revealed that camo_SRS_ was significantly correlated with both CAT‐Q score and camo_ToM_. There was no significant correlation between CAT‐Q and camo_ToM_ in the whole sample (See Table 2 for correlation coefficients).

**TABLE 2:** Nonparametric correlations between self‐reported and discrepancy camouflaging measures including the whole sample

	Camo_ToM_	Camo_SRS_
CAT‐Q Score	0.043	0.364***
Camo_ToM_		0.516***

*** *p* < 0.001.

When examining sex differences, a similar pattern was found for females and males; camo_SRS_ was significantly correlated to both CAT‐Q and camo_ToM_ (See Table 3 for correlation coefficients). Fishers *r* to *z* transformation revealed a significantly stronger association between camo_ToM_ and camo_SRS_ for males, than females, *z* = 1.67, *p* = 0.047.

**TABLE 3:** Correlations between self‐reported and discrepancy camouflaging measures; male data are shown in the top diagonal and female data are shown in the bottom diagonal

	CAT‐Q score	Camo_ToM_	Camo_SRS_
CAT‐Q Score		0.093	0.300*
Camo_ToM_	0.023		0.633***
Camo_SRS_	0.436***	0.407***	

* *p* < 0.05;

*** *p* < 0.001.

### Interaction and main effects of camouflaging measure, sex, and group

A 3 (camouflaging measure) * 2 (group) * 2 (sex) ANOVA revealed a nonsignificant three‐way interaction *F*(1.82, 205.67) = 0.39, *p* = 0.66, partial *η* ^2^ = 0.003.

There was a statistically significant two‐way interaction between camouflaging measure and group, *F*(1.82, 205.67) = 9.61, *p* < 0.001, partial *η* ^2^ = 0.078. Post hoc analysis revealed a main effect of group for camo_ToM_ (*F*(1, 113) = 10.34, *p* = 0.002, partial *η* ^2^ = 0.084) and camo_SRS_ (*F*(1, 113) = 11.62, *p* = 0.001, partial *η* ^2^ = 0.093) scores but not CAT‐Q (*F*(1, 113) = 1.34, *p* = 0.25, partial *η* ^2^ = 0.012). For both discrepancy measures (camo_ToM_ and camo_SRS_), HT participants scored significantly higher than Dx participants (mean difference 0.086 95% CI [0.033, 0.140], 0.091 95% CI [0.038, 0.143] for camo_ToM_ and camo_SRS_, respectively). The effects withstood Bonferroni correction.

**FIGURE 1:**
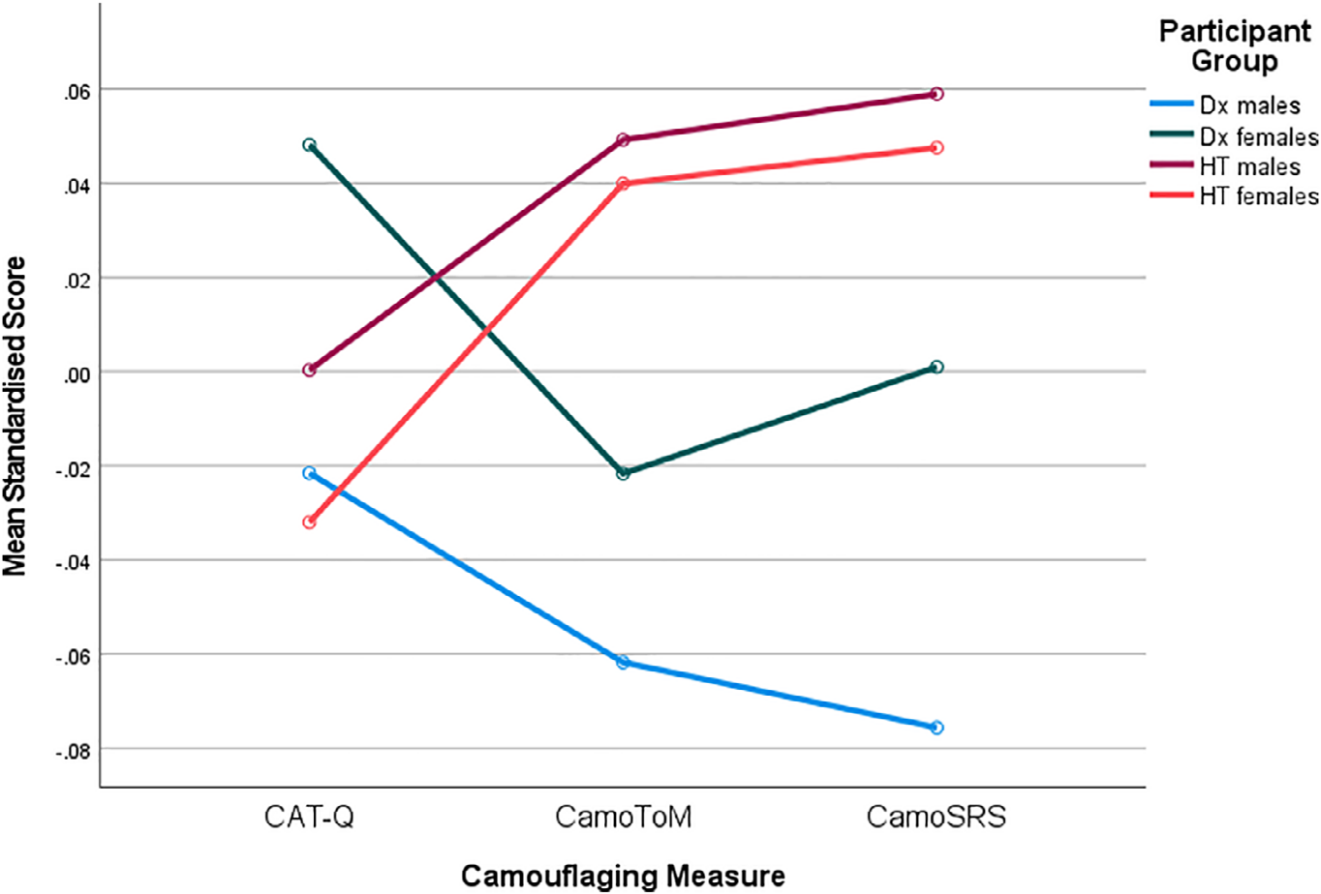
Mean scores across camouflaging measures and groups

There was also an overall significant two‐way interaction between group and sex, *F*(1, 113) = 4.34, *p* = 0.04, partial *η* ^2^ = 0.037. Examination of plots (see Figure 1) suggests Dx females scored higher than Dx males on all camouflaging measures, but no sex differences occurred in the HT group. Post hoc 2*2 ANOVAs were conducted for each camouflaging measure. There were no significant two‐way interactions between sex and group when examining each measure separately. Main effects were then explored.

For the CAT‐Q, there was no significant main effect of group or sex.

For camo_ToM_, there was a significant main effect of group (*F*(1, 153) = 13.39, *p* < 0.001), for the males (*F*(1, 153) = 9.71, *p* = 0.002) and females (*F*(1, 153) = 4.30, *p* = 0.04). For both sexes, HT participants scored significantly higher than Dx participants (mean difference 0.068 95% CI [0.003, 0.132] and 0.098 95% CI [0.036, 0.161] for females and males, respectively). However, this effect withstood Bonferroni correction for males only.

For camo_SRS_, there was a significant main effect of group (*F*(1, 124) = 14.75, *p* < 0.001). This effect was only significant for males (*F*(1, 124) = 16.71, *p* < 0.001). Pairwise comparisons revealed that HT males scored significantly higher than Dx males, mean difference = 0.143, 95% CI [0.074, 0.213]. This effect was maintained with Bonferroni correction. There was no significant difference between Dx and HT females, nor between sexes within each group (all ps > 0.05).

## DISCUSSION

This study aimed to compare three measures of camouflaging between males and females with an autism diagnosis, and those with equally high levels of autistic traits but no diagnosis. The two discrepancy scores correlated with each other, both within the whole sample and for males and females separately; likely driven, in part, by the fact that the ADOS‐2 contributes to each score. However, self‐reported camouflaging on the CAT‐Q correlated with the camo_SRS_ scores only. This finding suggests that discrepancy scores using self‐report measures of autistic traits may be capturing the same construct of camouflaging as CAT‐Q scores. These results could reflect that individuals who are consciously aware of their own autistic traits, are also more aware of their own camouflaging attempts. An alternative explanation is that items on the CAT‐Q may closely map onto difficulties outlined in autistic traits measures, and therefore the correlation may be driven by the correlation between both CAT‐Q and Camo_SRS_ with autistic traits. By contrast, the camo_ToM_ scores did not correlate with CAT‐Q self‐reported camouflaging. This may be due to camo_ToM_ scores measuring the effectiveness of deeper compensation strategies that are less overt or conscious, and thus, awareness of these strategies is possibly not reflected in self‐reported measures of camouflaging.

Interestingly, the relationship between the two discrepancy scores (camo_ToM_ and camo_SRS_) was significantly stronger for males than females. Previous evidence has demonstrated a strong link between ToM and self‐reported autistic traits (Ronald et al., 2006). One possible explanation for this current finding is a stronger relationship between self‐reported autistic traits (SRS scores) and ToM for males, compared with females. Emerging evidence of a “female autism phenotype” suggests that some autistic females may present their autistic features differently to autistic males (Hull et al., 2020). For instance, some autistic females may be more socially motivated than some autistic males (Sedgewick et al., 2016), therefore may not score highly on items reflecting preference for being alone or not enjoying social situations on the SRS. It may be, therefore, that the SRS is a more accurate tool for measuring autistic traits for males. However, this requires further examination that is beyond the scope of this article. Interestingly, females in this sample had significantly higher SRS scores compared with males. This could possibly be due to an increased awareness of their own traits due to later age of diagnosis (perhaps involving a longer diagnosis process (Begeer et al., 2013) although this cannot be determined from the available data), resulting in a more accurate reflection of their autistic traits. Future research should adopt a longitudinal approach to examine whether level of self‐reported traits, and/or camouflaging, changes subsequent to diagnosis, and whether this differs across males and females.

A significant group*measure interaction revealed that high trait participants scored higher than diagnosed groups on discrepancy measures of camouflaging, but not self‐reported camouflaging. This partially confirms our hypothesis. From these results we propose the interpretation that, although individuals with high autistic traits with and without a formal diagnosis may report camouflaging *intent* to a similar extent, *effective* camouflaging is more characteristic of those without a diagnosis. Camouflaging has been identified as a possible barrier to diagnosis for autistic females (Lockwood‐Estrin et al., 2021), however the current study adds potential evidence for effective camouflaging being a barrier for males with high autistic traits, who might also meet threshold for diagnosis were it not for their ability to camouflage their autistic traits (Lewis, 2017).

When examining the main effect of group on discrepancy measures of camouflaging, findings revealed that high trait males camouflaged significantly more than diagnosed males. The same pattern was not found for females in this sample. This suggests that diagnosed females and high trait females may be camouflaging with similar levels of effectiveness. It has been proposed that differences in camouflaging between males and females may be driven by sex‐specific sociocultural expectations and pressures (Kreiser & White, 2014); might an autism diagnosis give males, but not females, “permission” to show their autistic features? It is important to note that the lack of group*sex interaction within each measure suggests that the strength of the group effect was not significantly different. However, this possible difference between male and female groups deserves further empirical investigation in larger, representative samples.

There was no overall main effect of sex, thus refuting our hypothesis that females (in both groups) camouflage more than males (in both groups) across measures. However, within the three‐way ANOVA, a group*sex interaction was revealed across all camouflaging measures. Examination of plots suggests that, within the diagnosed group, females scored higher than males overall, whereas in the high trait group there was no sex difference. Similar findings were reported by Hull et al. (2019) who found diagnosed autistic females scored significantly higher on the CAT‐Q than autistic males, when controlling for autistic traits and age; however, no sex differences were found in the nonautistic group. Combined with the significant effect of group, this might indicate that when considering camouflaging as an overall concept (incorporating both intent and effective camouflaging), autistic males camouflage the least of our four groups. This overall conceptualization of camouflaging including both intent and effectiveness of camouflaging deserves further investigation, as understanding the differences may explain the inconsistent sex differences reported to date.

### Limitations

It is important to note the limitations of our study. Our sample size is fairly small when participants are separated into the four sex*diagnosis groups, therefore, the analysis may have been underpowered to detect differences. We also adopted a binary view of biological sex, without exploring nuances of other gender identities. If camouflaging is primarily a stigma driven strategy (Pearson & Rose, 2021), it could be that individuals identifying as a gender other than cis‐male/female adopt additional camouflaging strategies due to the intersectional nature of the stigma they may face. It is important to include gender diverse individuals in future research to examine the impact of camouflaging intent and effectiveness on their lives. Similarly, our sample was predominantly white; camouflaging experiences may be different for individuals with nonwhite heritage. It is not clear from this sample whether these findings would extend to those in other age groups. Furthermore, this study did not include individuals with additional learning disabilities or those who were nonverbal, for whom camouflaging measures have not yet been designed.

In addition, it is important to consider the validity of measures used to create the discrepancy scores in this study. The ADOS‐2 was used as it is currently the “gold standard” observer‐rated measure of autistic behaviors and is commonly used to facilitate diagnostic practice. However, there is some question as to whether this measure can validly capture individual differences in autistic traits, particularly for females (Hull et al., 2020). It is possible that some females may have different, nontraditional, presentations of autism that are not fully captured in the ADOS‐2 scoring (Wilson et al., 2016). This is potentially due to a male biased lens historically adopted in autism research, and an antiquated assumption that autism only, or predominantly, affects males. Discussing the limitations of the ADOS‐2 in detecting autism in females is beyond the scope of this article, however, it is important that future studies consider whether the discrepancy between observed autistic behaviors via the ADOS‐2 and self‐reported autism fully represent camouflaging, or instead the limits of the ADOS‐2 itself in detecting nontraditional autism presentations.

Furthermore, in this study we used ToM measures as a proxy for underlying cognitive difficulties reflecting greater autism. Autism is a heterogenous condition and adopting this approach does not account for other cognitive differences which may underlie other aspects of autism such as repetitive behaviors and intense interests. Future studies should attempt to examine the discrepancy between cognitive proxies of these characteristics and observed autistic behaviors.

In addition to considering the measure used when conceptualizing and operationalizing camouflaging in future studies, in particular when examining “effective” camouflaging, it is imperative that autistic voices are centered. For instance, it is important to clarify what is meant by “effective” camouflaging when including this concept in empirical studies. For one‐person, effective camouflaging could mean that their autistic characteristics are not identified by another person, whereas for another person, effective camouflaging might alternatively or additionally be defined by obtaining an external outcome such as gaining employment. These definitions will also impact the measures used when operationalizing effective camouflaging and should be considered further in future research.

### Implications

Despite the aforementioned limitations and considerations, the possible implications of this study span both research and clinical settings. First, it is important that future research examines the effectiveness of camouflaging strategies, rather than simply intent, when focusing on the potential consequences of masking or compensating for autistic behaviors. It could be that ineffective strategies have greater negative consequences due to the exertion of energy with fewer perceived benefits. Alternatively, individuals who implement effective strategies may exert greater energy, thus resulting in poorer wellbeing and quality of life. In addition, further investigation of factors that might influence the effectiveness of camouflaging is needed. When considering environmental influences, it could be that more structured home or school environments lead to more effective camouflaging strategies. Alternatively (or additionally), cognitive influences such as greater executive functioning ability or IQ may lead to more effective camouflaging strategies. Although some research has begun to examine this (e.g., Lai et al., 2017, Livingston et al., 2019), future studies are needed to further our understanding.

Second, it is important for future research to better establish the construct of camouflaging. In particular, there is a wider discussion about the best measures to use when adopting a discrepancy approach and the extent to which camouflaging intent and camouflaging effectiveness overlap (see, Williams, 2022).

Finally, our findings suggest that effective camouflaging may reduce the likelihood of autism diagnosis. Diagnosis can increase access to wider support and accommodations. This is essential as evidence suggests that timely access to support can improve outcomes and quality of life for autistic individuals (Atherton et al., 2021). An alternative view is that individuals with high levels of autistic traits, but no diagnosis, do not require additional support. However, to ensure that all autistic individuals receive the support they need, camouflaging strategies should be considered during the assessment process to ensure a timely diagnosis for all.

## CONCLUSION

To the best of our knowledge, this was the first study to examine sex differences comparing self‐report and discrepancy measures of camouflaging in individuals with high autistic traits with versus without a formal diagnosis. Results revealed that discrepancy measures of self‐reported autistic traits correlated with self‐reported camouflaging. Furthermore, the findings show that camouflaging intent (measured via self‐report measures) may be similar across males and females with and without a formal autism diagnosis; however, greater scores on discrepancy measures for the high trait group suggest that effective camouflaging may be a barrier to autism diagnosis for individuals with high autistic traits. Finally, diagnosed females were found to camouflage more across all measures than diagnosed males, whereas no sex differences were found within the high trait sample. This may be driven by historical male stereotypes of autism and consequently, reduced societal acceptance of autism characteristics for autistic females. These findings highlight several areas for future research and improvements to diagnostic processes to ensure timely diagnosis for all, and to address possible negative outcomes associated with camouflaging.

## CONFLICT OF INTEREST

The authors have no conflicts of interest to declare that are relevant to the content of this article.

## ETHICS STATEMENT

Ethical approval was obtained from Bromley NHS Research Ethics Committee (reference number: 16/LO/1472).

## Supporting information


**APPENDIX S1:** Supporting Information

## Data Availability

The data that support the findings of this study are available on request from the corresponding author. The data are not publicly available due to privacy or ethical restrictions.
