# Supplementary material for: A comparison of self‐report and discrepancy measures of camouflaging: Exploring sex differences in diagnosed autistic versus high autistic trait young adults
Source: Autism Res. 2022 Dec 9;16(3):580–90. doi: 10.1002/aur.2873 (PMC10946751; doi:10.1002/aur.2873)
Supplement: Supplementary file 1 — APPENDIX S1: Supporting Information [file AUR-16-580-s001.docx]

**Supplementary Materials**

**The Social Relationships Study – Phase Three**

***Recruitment Streams for Diagnosed Autistic and High Autistic Trait Participants***

TEDS Twin with an Autism Diagnosis from the first phase of the SRStudy

N= 129

TEDS Twins who had high scores (>12) on the Childhood Autism Spectrum Test (CAST) collected at aged 8 and/or 12 years

N= 552

Recruited into diagnosed male group

N= 46

Recruited into diagnosed female group

N= 40

Twins already in SRStudy removed

N=123;

remaining N=429

Twins who’s CAST score reduced between aged 8 and 12 removed

N=72;

remaining N=357

**Diagnosed Sample**

**High Trait Sample**

Twins who have withdrawn from TEDS removed

N=22;

remaining N=335

Twins with a TEDS record of a “late” diagnosis of autism (since SRS-1)

N=1;

remaining N=334

Recruited into high trait male group

N= 45

Recruited into high trait female group

N= 43

Note: A priori power analysis determined a minimum sample size of n=40 for each group to detect moderate (0.5) group effects.

All participants from the possible samples were contacted and invited to take part in the study (n=129 diagnosed participants, n=334 high trait participants).

The final samples reflect the first participants who gave consent to taking part in the study. Data were collected between 2016 – 2019.

**The distribution, spread and average of standardised self-reported and discrepancy scores of camouflaging.**


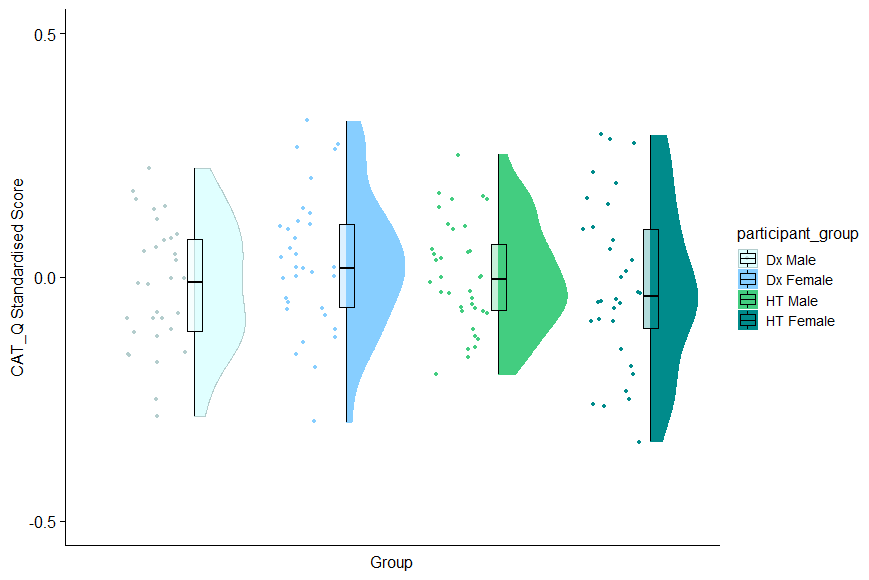


*Figure 3: Raincloud plot showing distribution and mean score on the CAT_Q for each group. Dx = Diagnosed, HT = high trait*


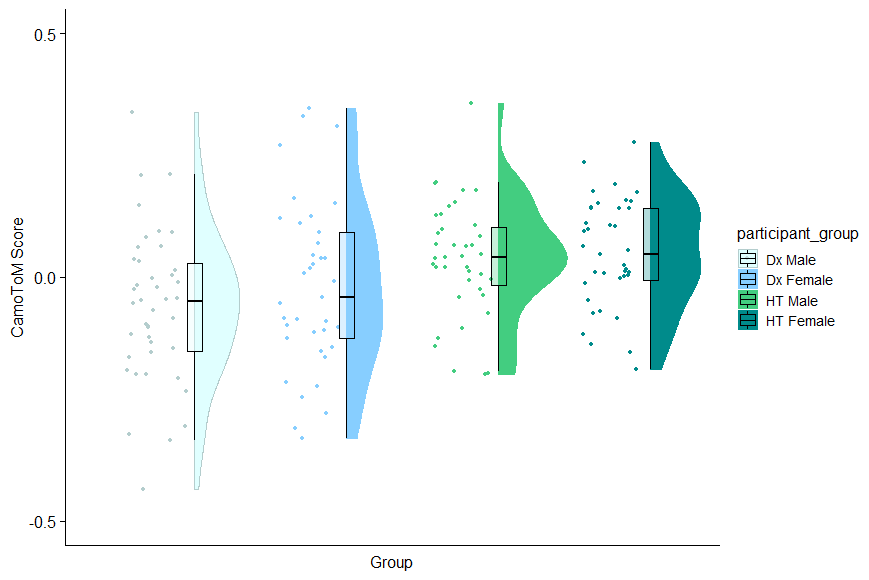


*Figure 4: Raincloud plot showing distribution and mean score on the Camo_ToM_ for each group. Dx = Diagnosed, HT = high trait*


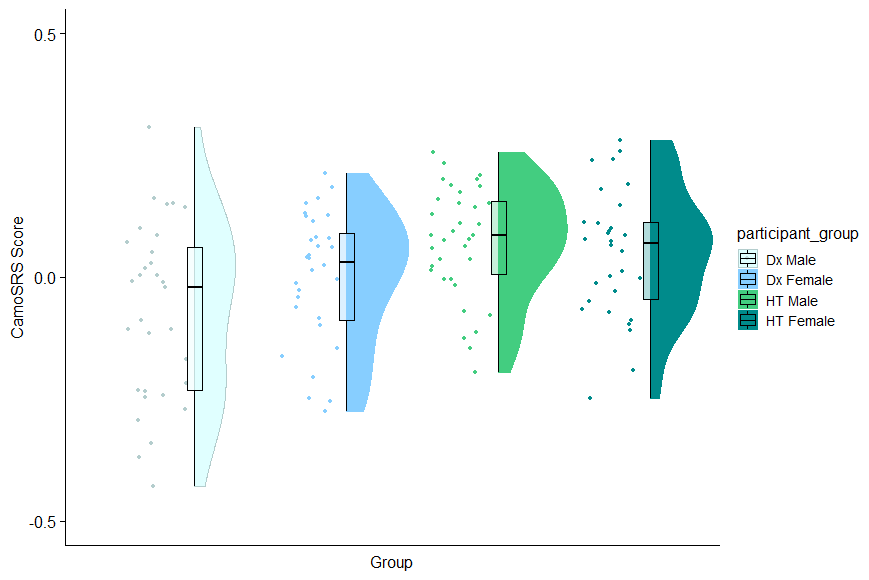


*Figure 5: Raincloud plot showing distribution and mean score on the Camo_SRS_ for each group. Dx = Diagnosed, HT = high trait*
